# Chondroitin Sulfate Protects the Liver in an Experimental Model of Extra-Hepatic Cholestasis Induced by Common Bile Duct Ligation

**DOI:** 10.3390/molecules27030654

**Published:** 2022-01-20

**Authors:** Pedro L. R. Guedes, Carolina P. F. Carvalho, Adriana A. F. Carbonel, Manuel J. Simões, Marcelo Y. Icimoto, Jair A. K. Aguiar, Maria Kouyoumdjian, Marcos L. Gazarini, Marcia R. Nagaoka

**Affiliations:** 1 Department of Medicine, Escola Paulista de Medicina, Universidade Federal de São Paulo, São Paulo 04023-062, Brazil; guedesffb@gmail.com; 2 Department of Biosciences, Instituto Saúde Sociedade, Universidade Federal de São Paulo, Santos 11015-020, Brazil; ccarvalho13@unifesp.br (C.P.F.C.); marcos.gazarini@unifesp.br (M.L.G.); 3 Department of Gynecology, Escola Paulista de Medicina, Universidade Federal de São Paulo, São Paulo 04039-001, Brazil; adricarbonellfisio@hotmail.com; 4 Department of Morphology and Genetic, Escola Paulista de Medicina, Universidade Federal de São Paulo, São Paulo 04023-900, Brazil; mjsimoes@unifesp.br; 5 Department of Biophysics, Escola Paulista de Medicina, Universidade Federal de São Paulo, São Paulo 04039-032, Brazil; marceloicimoto@gmail.com; 6 Department of Biochemistry, Universidade Federal de Juiz de Fora, Juiz de Fora 36036-900, Brazil; jair.aguiar@ufjf.edu.br; 7 Department of Biochemistry, Escola Paulista de Medicina, Universidade Federal de São Paulo, São Paulo 04023-062, Brazil; mkouyoumdjian@unifesp.br

**Keywords:** liver, fibrosis, chondroitin, regeneration, apoptosis, inflammation

## Abstract

During liver fibrogenesis, there is an imbalance between regeneration and wound healing. The current treatment is the withdrawal of the causing agent; thus, investigation of new and effective treatments is important. Studies have highlighted the action of chondroitin sulfate (CS) in different cells; thus, our aim was to analyze its effect on an experimental model of bile duct ligation (BDL). Adult Wistar rats were subjected to BDL and treated with CS for 7, 14, 21, or 28 days intraperitoneally. We performed histomorphometric analyses on Picrosirius-stained liver sections. Cell death was analyzed according to caspase-3 and cathepsin B activity and using a TUNEL assay. Regeneration was evaluated using PCNA immunohistochemistry. BDL led to increased collagen content with corresponding decreased liver parenchyma. CS treatment reduced total collagen and increased parenchyma content after 21 and 28 days. The treatment also promoted changes in the hepatic collagen type III/I ratio. Furthermore, it was observed that CS treatment reduced caspase-3 activity and the percentage of TUNEL-positive cells after 14 days and cathepsin B activity only after 28 days. The regeneration increased after 14, 21, and 28 days of CS treatment. In conclusion, our study showed a promising hepatoprotective action of CS in fibrogenesis induced by BDL.

## 1. Introduction

Liver fibrosis is a common response to chronic liver damage, caused mainly by chronic hepatitis C infection, alcohol, and non-alcoholic steatohepatitis. The end stage is cirrhosis, with clinical complications including liver failure, portal hypertension, and hepatocellular carcinoma [1]. Fibrosis and cirrhosis represent a massive care burden worldwide. In 2017, chronic liver diseases caused over 1.3 million deaths worldwide [2].

One of the many agents involved in the etiopathogenesis of liver diseases is biliary tract obstruction. Cholestasis results in a high concentration of bile in hepatocytes, which may lead to apoptosis in vivo and in vitro, mediated by activation of death receptors such as Fas, TNF receptor 1, and TRAIL, which are largely expressed in liver cells [3,4]. Recruited neutrophils and the secretion of reactive oxygen species participate in the activation of NF-κB, which in turn promote the increase in pro-inflammatory cytokines, which also induce oxidative stress in a stimulating cycle perpetuating the hepatic injury [5,6].

Extracellular matrix (ECM) changes are related to the secretion of cytokines (pro-fibrogenic and pro-inflammatory) and matrix metalloproteinases, leading to hepatic stellate cell activation and differentiation to the profibrogenic phenotype. These events are responsible for the degradation and renewal of the ECM, which may contribute to the pathogenesis of the initial stages of liver fibrosis [7,8].

Hepatic cell death and consequent loss of parenchyma cause an increase in metabolic demand and extensive functional deficit. In this context, hepatocellular regeneration may eventually restore hepatic architecture and function, as observed in an experimental partial hepatectomy model [9,10]. Despite being considered in acute hepatic diseases, liver regeneration has been highly associated with recovery from fibrosis in chronic liver diseases [11].

The experimental model of bile duct ligation (BDL) has been used to induce extra-hepatic cholestasis in rats, promoting liver injury, intra-hepatic epithelial biliary cell proliferation, myofibroblastic differentiation of hepatic stellate cells, and ECM deposition [12,13]. Even with all the knowledge of hepatic fibroproliferative diseases, the current treatment of liver fibrosis is limited to removal of the injurious stimuli, control of biological mediators of inflammation and fibrogenesis, and ultimately liver transplantation since there are no direct antifibrotic therapies available to date [14]. Therefore, there is an urgent need to develop innovative methods of treatment.

Chondroitin sulfate (CS) is a polysaccharide from ECM with substantial structural variability in terms of size and sulfate position and degree due to biosynthetic changes [15]. The high density of negative charges due to sulfation and carboxyl groups and many functional domains of CS allow it to interact with several molecules of the ECM [16]. CS has been reported to play a role in many important biological functions, such as inflammation, cell proliferation, dedifferentiation, migration, tissue morphogenesis, organogenesis, infection, and tissue repair [17,18], and can be considered a compound with a potential protective effect on pathological situations. In the liver, its effect on oxidative stress in an experimental model of CCl_4_-induced hepatotoxicity was demonstrated [19]. Thus, this study aimed to evaluate the hepatoprotective effect of CS in experimental BDL-induced liver fibrogenesis.

## 2. Results

### 2.1. Characterization of the Animals Subjected to BDL Model

The experimental model of liver fibrosis was successfully developed in all BDL animals. Sham animals did not develop fibrosis, and their results are presented only for comparison of the caspase and cathepsin B activity with BDL groups.

Table 1 shows the descriptive parameters of BDL and BDL-CS groups in all studied times (7, 14, 21, and 28 days).

The age of the animals in the BDL-14-day group was higher (*p* < 0.04) than those from 7 days. In the treated group, the age in the BDL-CS-14-day group was lower (*p* < 0.0007) than the corresponding non-treated group (BDL-14d). In addition, BDL-CS-28d had a higher age compared to the BDL-CS-7d (*p* < 0.03) and BDL-CS-14d (*p* < 0.04) groups.

Concerning body weight, both BDL and BDL-CS groups had a progressive increase in body weight (BW, g) over the studied period, stabilizing after 21 and 28 days. In the BDL group, an increase (*p* < 0.04) in the BW after 14, 21, and 28 days compared to the 7-day group was observed. In the BDL-CS group, we observed a significant decrease (*p* < 0.0001) in the BW after 21 and 28 days of treatment when compared to BDL-CS-7 and 14-day groups. The BDL-CS-14d group had lower BW (*p* = 0.0005) compared to the corresponding non-treated group.

In relation to liver weight (LW), we observed that LW from both BDL and BDL-CS groups increased (*p* < 0.0001) compared to corresponding sham groups, as a consequence of the fibrogenesis process (data not shown). In addition, we observed a significant increase (*p* < 0.0001) in LW over the time studied in the BDL group compared to the 7-day group. For the BDL-CS group, we also observed a significant increase but only at 21 and 28 days, compared to the 7- and 14-day groups. LW of the BDL-CS 14-day group was lower (*p* = 0.0072) compared to the untreated BDL 14-day group.

The liver/body weight relation (L/BW) (%) was significantly higher (*p* = 0.0172) after 21 days of induction (BDL-21d) than after 7 days (BDL-7d group). In the treated group, it was verified that L/BW was also higher (*p* < 0.0001) after 21 days but also after 28 days of induction versus that of 7 days. In addition, L/BW of BDLCS-28d was significantly higher (*p* = 0.0054) than the BDL-CS-14d group. Although we observed these differences, the L/BW was not different when we compared each BDL-CS group to its corresponding BDL group.

The hepatocellular injury was assessed by serum ALT and AST levels and by histological analysis of hematoxylin-eosin (HE)-stained-livers. In relation to serum ALT or AST, we did not observe any significant difference among the groups, although a slight decrease was noticeable in most of the BDL-CS groups compared to their corresponding BDL groups. Histopathological analysis of the HE stained-BDL livers (Figure S1A–H, see Supplementary Materials) showed progressive disorganization of the cytoarchitecture with a porto-portal bridging formation, the proliferation of periportal biliary ductules, and areas of polymorphonuclear infiltration. After 7 days of BDL surgery (Figure S1A,B), it was possible to observe an important bile ductular proliferation and infiltration of inflammatory cells. At 14 days of BDL induction (Figure S1C,D), there was a marked increase in the portal triads areas as a consequence of the bile ductular proliferation, but the formation of interductular connective tissue was also evident. After that, we observed the porto-portal bridges forming fibrous septa at 21 (Figure S1E,F) and 28 days with compression of the parenchyma more evident in the BDL-untreated group (Figure S1G) than in the BDL-CS group ((Figure S1H).

### 2.2. Chondroitin Sulfate Improves Liver Fibrogenesis

**Figure 1:**
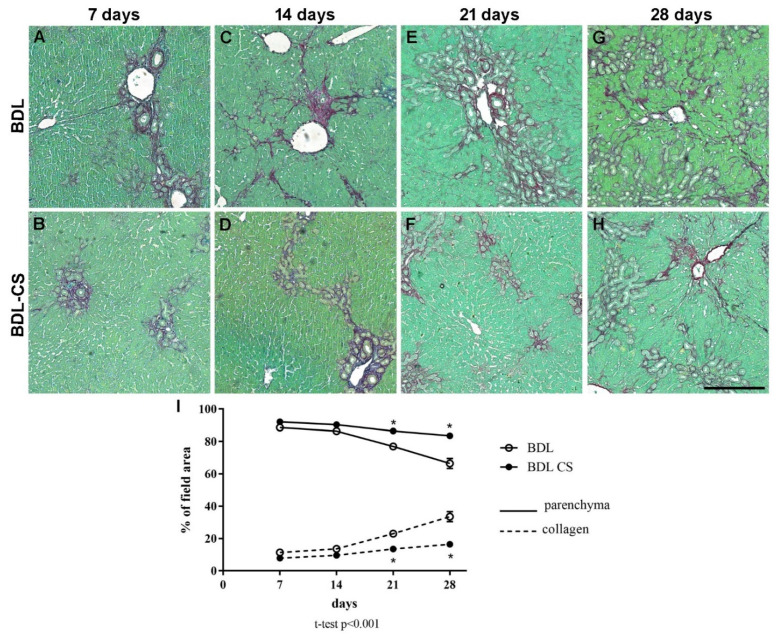
Liver histology and quantification of fibrogenesis of BDL animals treated with vehicle (BDL group) or chondroitin sulfate (BDL-CS group). (**A**–**H**) Representative images of the Picrosirius-stained liver sections from BDL and BDL-CS groups (100× magnification). (**I**) Histomorphometric analysis of the liver parenchyma and collagen contents (% of field area) from BDL and BDL-CS animals. Values are expressed as mean ± SEM, *n* minimal of 4 animals/group. * *p* < 0.001, multiple *t*-test. Scale bar: 250 µm.

Fibrogenesis induced by BDL led to changes to portal triads and the progressive appearance of fibrotic septa as a function of the induction time (Figure 1).

Figure 1A–H shows representative images of PS-stained liver sections from BDL (vehicle-treated groups) and BDL-CS groups (CS-treated groups) at 7, 14, 21, and 28 days after surgery and treatment.

Panel I shows histomorphometric quantification of collagen and parenchyma contents in liver sections from different groups. As expected, we observed a progressive increase in collagen content and a corresponding decrease in liver parenchyma as a function of time in the BDL group (Figure 1I). In the BDL-CS group, we observed a small increase in collagen content (% of field area) after 21 days (14 ± 1%) and 28 days (16 ± 2%), which were significantly smaller (*p* < 0.0001) than those of the corresponding BDL groups (21 days: 23 ± 1% and 28 days: 34 ± 3%).

Conversely, in relation to parenchyma content (% of field area), we observed a progressive decrease during the induction time in the BDL group (Figure 1I), especially after 21 (77 ± 2) and 28 days (67 ± 3), which were significantly different (*p* < 0.0001) after CS treatment (21 days: 86 ± 1 and 28 days: 84 ± 2).

### 2.3. Collagen Fibers Organization in the Hepatic Veins after Chondroitin Sulfate Treatment

**Figure 2:**
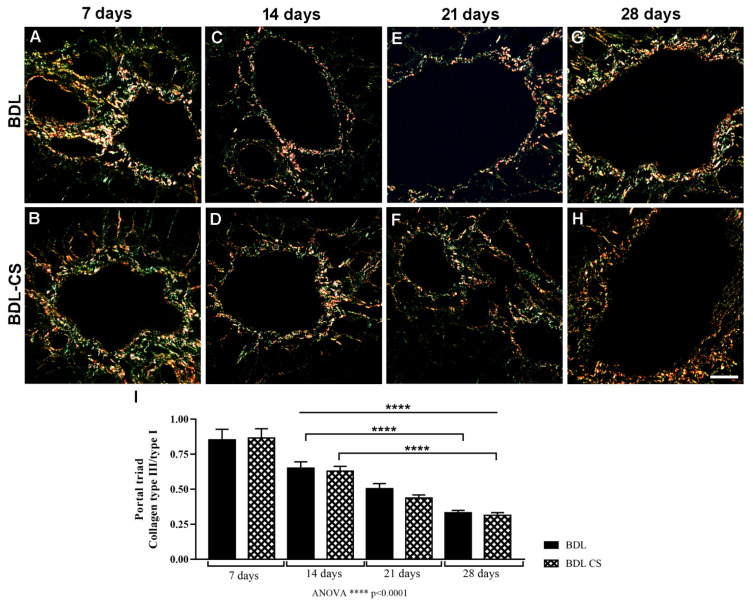
Collagen fibers organization in the portal triads of picrous–sirius (PS)-stained liver sections from BDL rats treated with vehicle (BDL group) or chondroitin sulfate (BDL-CS group) analyzed by polarized light microscopy. (**A**–**H**) Representative images of portal triads in polarized light-analyzed liver sections from BDL and BDL-CS groups (200× magnification), with type I (yellow and red) and type III (green) collagens. (**I**) Collagen type III/type I relation in the portal triads from liver sections from BDL and BDL-CS groups. Values are expressed as mean ± SEM, (*n* = 4 animals/group). ANOVA with Tukey post hoc test: Scale bar: 400 µm.

The collagen fibers organization in the liver fibrogenesis was analyzed in the regions of centrilobular veins and portal triads from PS-stained liver sections by polarized light microscopy, where it was possible to differentiate type I (yellow and red) and type III (green) collagen fibers. In the centrilobular veins, no difference was observed between both collagen contents in relation to the time of induction or the treatment. However, in the portal triads, differences were found, and Figure 2A–H shows the representative images from PS-stained liver sections of the BDL and BDL-CS groups at 7, 14, 21, or 28 days after surgery, and treatment was observed with a polarized light microscope. The relation of collagen III to collagen I content found in portal triads from BDL and BDL-CS groups (Figure 2I) decreased over time of induction and significantly (*p* < 0.0001) compared to the corresponding 7-day groups. In addition, this relation was lower (*p* < 0.0001) in the BDL and BDL-CS groups after 28 days compared to the respective groups after 14 days.

### 2.4. Chondroitin Sulfate Decreases Cell Death

**Figure 3:**
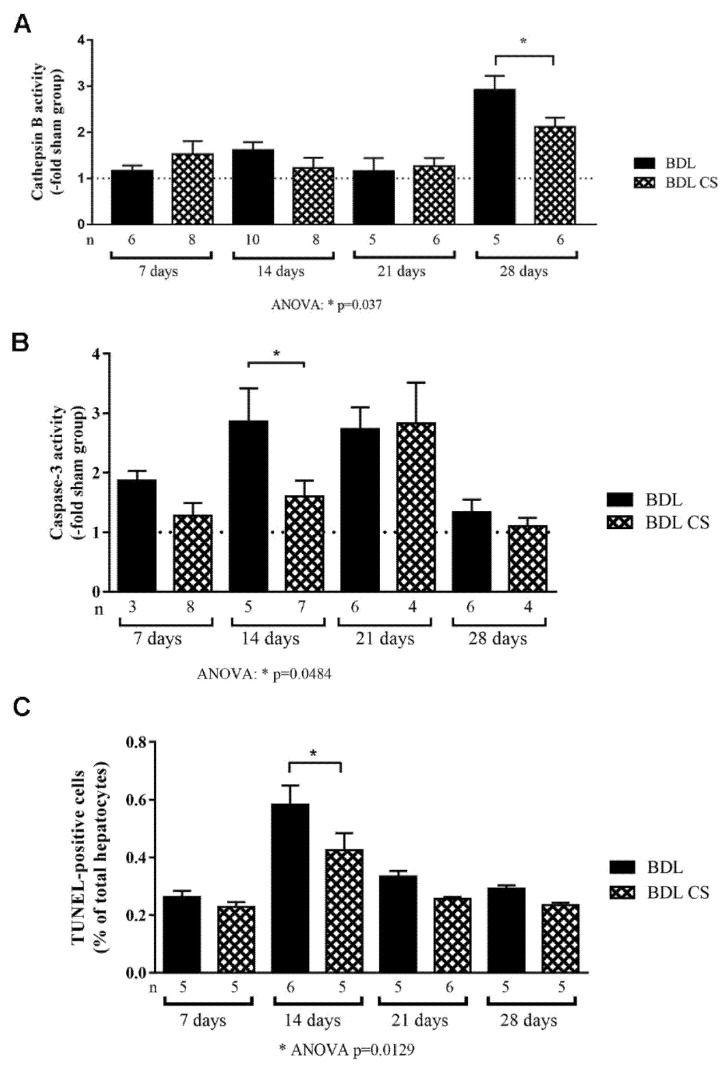
Cell death pathway analysis in the liver homogenates from BDL animals treated with vehicle (BDL group) or chondroitin sulfate (BDL-CS group). Enzymatic activity of cathepsin B (**A**) and caspase-3 (**B**) in the liver homogenates (fold-increase compared to the sham group). Panel (**C**) shows the quantification of TUNEL-positive hepatocytes in the liver from BDL or BDL-CS groups at 7, 14, 21, and 28 days after induction. Values are expressed as mean ± SEM. * ANOVA with Tukey post hoc test.

We explored the cell death pathway during the fibrogenesis process by measuring cathepsin B and caspase-3 activity in the liver homogenates (Figure 3A,B, respectively). During the first 21 days of BDL induction, we did not observe a significant increase in hepatic cathepsin B activity, expressed as a fold-increase in relation to the sham group, in either control (BDL) or CS-treated (BDL-CS) groups. However, after 28 days of CS treatment, the cathepsin B activity decreased significantly (*p* = 0.037) when compared to the corresponding group without treatment (Figure 3A).

In Figure 3B, we can observe that in the BDL group, caspase-3 activity increased after 7, 14, and 21 days of induction, followed by a reduction of activity after 28 days, reaching sham levels. In the BDL-CS group, we observed a peak of activity after 21 days of treatment. CS was able to significantly decrease (*p* = 0.0484) caspase-3 activity after 14 days of treatment when compared to the untreated group.

Hepatocellular apoptosis was confirmed after counting TUNEL-positive hepatocytes in the liver sections from BDL animals (Figure 3C). All BDL groups (treated or not) had few apoptotic cells (under 1% of total hepatocytes per field), although non-treated cholestatic animals presented a significant increase (*p* < 0.001) in the number of TUNEL-positive hepatocytes (0.7 ± 0.1%) after 14 days of induction when compared to other BDL groups. CS significantly reduced (*p* = 0.0129) the number of TUNEL-positive hepatocytes (0.5 ± 0.1%) in the liver after 14 days of induction in comparison with the corresponding group (BDL 14d) (Figure 3C).

### 2.5. Chondroitin Sulfate Promotes Hepatocyte Proliferation

**Figure 4:**
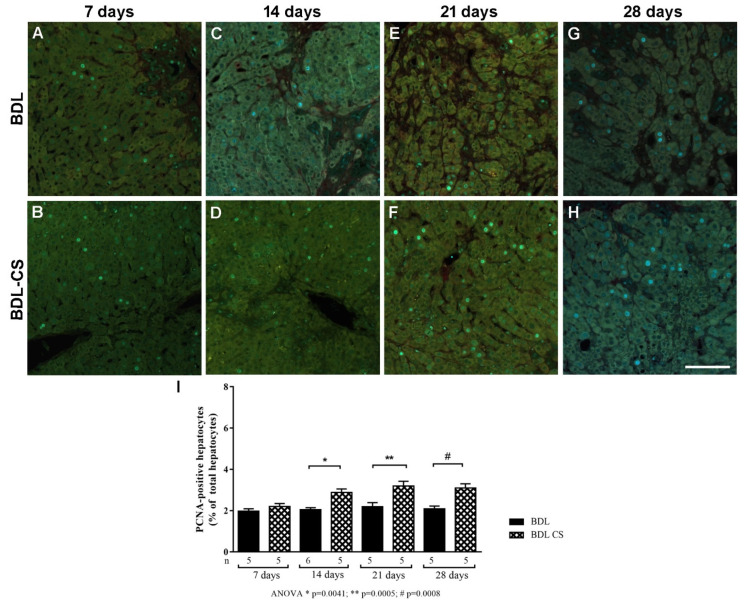
PCNA-positive hepatocytes in the BDL-liver of rats subjected to different periods of treatment with vehicle (BDL group) or chondroitin sulfate (BDL-CS group). Representative images of PCNA immunohistochemistry in the liver sections from BDL rats treated with vehicle (upper line) or CS (bottom line) for 7 (**A**,**B**), 14 (**C**,**D**), 21 (**E**,**F**), and 28 days (**G**,**H**). (**I**) Percentage of PCNA-positive hepatocytes quantified in relation to the total number of hepatocytes of field. Values are expressed as mean ± SEM. *ANOVA with Tukey post hoc test. Scale bar: 50 µm.

Liver regeneration was evaluated by immunohistochemistry of proliferating cell nuclear antigen (PCNA). Figure 4A–H shows a representative gallery of microphotographs from each studied period in both groups, BDL and BDL-CS. Figure 4I presents the quantification of PCNA-positive hepatocytes, expressed as a percentage of the total number of hepatocytes of the field. Note an increase in positive proliferative cells after 14, 21, and 28 days of CS treatment compared to corresponding BDL groups.

## 3. Discussion

Proteoglycans are one of the most important components of liver ECM, mainly constituting glycosaminoglycans (GAGs), dermatan sulfate (DS), heparan sulfate (HS), and minor amounts of CS [18]. In our previous study, we observed a change in the composition of the ECM in BDL-induced fibrosis, with an increase in DS and HS content after BDL [17]. Since CS is a molecule naturally present in the body, its supplementation is widely used, especially in the treatment of osteoarthritis due to its anti-inflammatory effect. In the present work, we present the beneficial effects of chronic treatment with CS in the fibrogenesis induced by BDL, which increased parenchyma content and decreased septa size.

The BDL model was successfully induced, and the fibrogenesis was accompanied by changes in different parameters such as body and liver weights, which were already reported [20]. In addition, the liver/body weight ratio increased in all BDL groups in relation to sham groups (data not shown) and over the time of induction, which indicates the development of the fibrotic process [21]. Some differences in the body and liver weight of the 14 days groups (BDL and BDL-CS) may be related to the difference in the age of animals of these groups. Because of these differences, the results were presented in relation to the hepatic protein.

The hepatocellular injury was analyzed according to the serum ALT and AST activity, and we found an increase in both enzymes in BDL groups compared to sham groups (data not shown) but no difference in relation to the CS treatment or the time of induction. These findings are in accordance with the literature, as it has been shown that the levels of aminotransferases reach a peak in the first 30–48 h and then decrease, although they remain higher than those in sham animals [22,23].

The hepatocellular injury and the progression of the fibrogenesis in the BDL model were followed by hematoxylin-eosin-stained liver sections, where the well-described histological alterations were observed, such as the proliferation of epithelial biliary cells, with major changes to portal triad and formation of fibrotic septa between portal spaces, a process called ductular reaction in BDL animals [12, 22]. Ductular reaction and ECM deposition are responsible for the major changes in the liver, resulting in the formation of fibrotic septa, the first sign of tissue fibrosis [8]. Many authors described liver fibrogenesis in BDL animals after 5 days of induction [23,24]. Using Picrosirius staining, a well-established method for evaluation of collagen content of both normal and fibrotic tissues [25], we performed a morphometric analysis discriminating parenchyma and septa. We observed that BDL led to a progressive and significant increase in collagen content, especially from the 14th to 28th days, accompanied by a decrease in hepatic parenchyma. CS treatment was able to slow the progression of fibrosis, mainly after 21 and 28 days of BDL induction, with preservation of liver parenchyma and reduction of septa formation.

It is well known that a mixture of collagens, especially I and III, comprise the liver tissue, which under normal conditions, have similar amounts and can correspond to 80% of the total liver collagen. In liver fibrosis, all types of collagens increase, but type I increases at a greater rate than type III [26]. Using the analysis by polarized light microscopy to differentiate these two types of collagen [27,28], we could observe the described profile in the portal triads, with an increased amount of collagen type I overtime of induction, but not in type III in accordance with previous studies [29,30]. These results demonstrate the rearrangement of the hepatic ECM in the portal triads during liver fibrogenesis, and CS did not interfere with it, although it had an important effect on reducing total collagen as mentioned before. The limitation of the collagen analysis by polarized light microscopy was the sensibility to differentiate the types I and III collagen in the septa, which did not allow for evaluation of the effect of CS in the collagen organization in those areas.

Along with other factors, the increased collagen content in the liver fibrogenesis contributed to an increase in intrahepatic vascular resistance, resulting in the development of portal hypertension, which is a critical factor for complications in advanced fibrosis [31]. Recently, it has been shown that the collagen measurement correlates to the portal pressure in animal models of fibrosis, whereas in patients, it correlates to the hepatic venous pressure gradient [31]. Thus, the collagen content is still one indirect method for monitoring the stage of liver fibrosis and can be used as a prognostic tool to manage the therapeutic strategy.

During liver fibrogenesis, there is an imbalance between liver regeneration and the wound healing process that involves mainly inflammation, cell death, and regeneration processes to reestablish homeostasis [32]. We analyzed the cell death process according to cathepsin B and caspase-3 enzymatic activity and with the TUNEL assay, and low activity (compared to sham) of cathepsin was observed up to 21 days after BDL surgery, followed by an increase on the 28th day. CS was able to decrease cathepsin activity but only after 28 days of treatment [33,34]. Caspase-3 activity was higher at 14 and 21 days of BDL and then decreased, as presented by other authors [35,36]. CS was able to significantly decrease caspase-3 activity after 14 days of treatment, which might be directly related to diminished hepatocellular apoptosis [33,35]. The relation between cathepsin B and caspase-3 activation in apoptosis was demonstrated in a bile salt-induced apoptosis model, in which caspase-3 could be considered as an initiator protease and cathepsin B a machinery protease, participating in an important degradation process [33].

The effect of CS on caspase-3 activity was also corroborated by the TUNEL assay, in which it was observed that CS treatment decreased the number of positive hepatocytes in the 14-day group. Inhibition of cell death in the BDL model was also observed when BDL mice were treated with pan-caspase inhibitor for 3 days, and reduced expression of effector caspases in hepatocytes was observed [35]. This effect was accompanied by a diminished expression of inflammatory chemokines, such as MIP-2, and lower HSC activation, which in turn reduced collagen deposition visualized by PS-stained liver specimens. Thus, in the extra-hepatic cholestasis model, the hepatic injury caused by caspase was pro-fibrogenic [35]. The failure in biliary salt secretion results in the high concentration of hepatocytes, which may induce apoptosis in vivo and in vitro after activation of cell death receptors, such as Fas and TRAIL [37,38,39], which was confirmed using Fas-deficient mice subjected to BDL that presented lower caspase-3 activity and, therefore, diminished hepatocellular death by apoptosis [40].

Apoptotic bodies are then engulfed by Kupffer cells, causing progression of injuries by stimulating the generation of death ligands and cytokine expression, thus promoting tissue inflammation, which induces production of ECM molecules by portal fibroblasts, contributing to fibrogenesis of cholestatic injuries [41,42]. Meanwhile, there are pathological changes to ECM due to an imbalance between MMPs and tissue inhibitors (TIMPs), which dictates worsening or improvement of this process [43].

The pharmacological activity of CS has been reported in different processes. The effect of GAGs was studied in a primary culture of chondrocytes stimulated by LPS [44], and it was observed that GAGs, such as hyaluronic acid, HS, and CS, were able to improve cell survival and modulated LPS-induced inflammation. This effect was mediated by inhibition of NF-kB nuclear translocation and reduced pro-inflammatory cytokine expression and caspase-3 activation [44]. Reduced translocation of this transcriptional factor was also observed in a CS-treated astrocyte culture stimulated by LPS, associated with a diminished expression of TNF-α and iNOS, indicating a possible neuro-immunomodulatory action of CS in neurotoxic conditions [45]. Nevertheless, the mechanisms involved in this process and the effects of CS on the pathophysiology of other diseases are not completely known [46].

The liver reacts to an insult, promoting a regeneration process that can lead to inflammation and finally cell death. However, if the insult is chronic, the tissue will need to be repaired, which causes excessive scarring, i.e., fibrosis and impaired liver function [47]. Normally, the slow turnover of hepatic cells is observed, but during the regeneration process, hepatocytes are the main cells involved. Conversely, during BDL-induced fibrogenesis, hepatocytes remain senescent, whereas ductular cells are the main cells involved in the proliferation process [47].

Liver regeneration capacity as a recovery tool after injury stimuli has been studied for many years [48,49,50]. Partial hepatectomy is the main experimental model used, although it does not reflect all liver diseases since the remaining tissue is normal in contrast to liver chronic diseases, where inflammation, cell death, and fibrosis are observed [51]. One of the used markers to evaluate hepatocyte proliferation is the proliferating cell nuclear antigen (PCNA), which is involved in vital cellular processes such as chromatin remodeling, DNA repair, sister-chromatid cohesion, and cell cycle control [52]. Analysis of liver regeneration according to PCNA expression in BDL animals has been extensively described [53,54,55,56]. In the present work, cholestasis led to the proliferation of epithelial biliary cells (data not shown) and hepatocytes; CS was able to increase hepatocyte proliferation after 14, 21, and 28 days of treatment.

The involvement of GAGs in hepatocyte proliferation was first described in 1979, when increased CS content in the ECM of hepatectomized rats was observed [57], with a similar pattern found in the liver of young animals [58]. Recently, it has been demonstrated that CS and DS may interact with heparin-binding (HB) proteins and HB-binding epidermal growth factor-like growth factor (HB-EGF), key factors for hepatocyte proliferation and antifibrosis in the BDL model [59,60,61], suggesting a protective role of these GAGs.

Although, in the past decades, the time course of fibrosis development has been well-established and many antifibrotic strategies have been experimentally tested, few approaches have become effective forms of antifibrotic therapy [62]. One reason is the difference between the experimental and human fibrosis development, which occurs over decades in humans. In addition, in humans, the early phase of fibrosis is often asymptomatic. Earlier treatment of fibrosis leads to more efficient regression. Therefore, antifibrotic therapy must be safe [62]. In the presented data, we show for the first time the hepatoprotective effect of exogenous CS on an experimental model of extrahepatic cholestasis using BDL in rats. Recently, the use of GAGs in tissue engineering systems has been described, especially for CS and hyaluronic acid, which can be used alone or in combination [63]. In conclusion, treatment with CS reduced fibrotic septa without changing the type III to I collagen ratio in the portal triads, and it decreased hepatocellular apoptosis, whereas it promoted liver regeneration, which indicates that this compound is a potential alternative to treating indications of cholestasis and retarding fibrogenesis and hepatic cirrhosis.

## 4. Materials and Methods

### 4.1. Animals

Male Wistar rats (6 to 8 weeks; *n* = 59, from CEDEME—Federal University of Sao Paulo) were housed (5 animals per cage) in a room maintained at 23 ± 1 °C with 12 h light/dark cycles and had free access to standard chow Nuvilab CR-1^®^ (Quimtia, Colombo, PR, Brazil) and filtered tap water. The basic composition of the chow was ground whole corn, soybean bran, wheat bran, salts (calcium carbonate, dicalcium phosphate, sodium chloride), vitamins (A, D3, E, K3, B1, B2, B6, B12, niacin, calcium pantothenate, folic acid, biotin, choline) and minerals (iron, manganese, zinc, copper, iodate, selenite, cobalt), amino acids (lysine, methionine) and additive (BHT, butylated hydroxytoluene). This study was approved by the Ethics Committee on the Use of Animals of the Federal University of Sao Paulo (protocol number 8415200314) and carried out according to the *Guide for the Care and Use of Laboratory Animals* [64].

### 4.2. Common Bile Duct Ligation (BDL) and Treatment

All surgical procedures were performed under inhalational anesthesia involving 1.5% isoflurane (Baxter, São Paulo, SP, Brazil), nitrous oxide (0.8 L/min), and oxygen (0.4 L/min) with clean surgical techniques as previously described [65]. Briefly, obstructive jaundice was induced by midline laparotomy and common bile duct exposure followed by double ligation and section between stitches. Sham-operated animals were subjected to a similar procedure, without common bile duct ligation.

**Figure 5:**
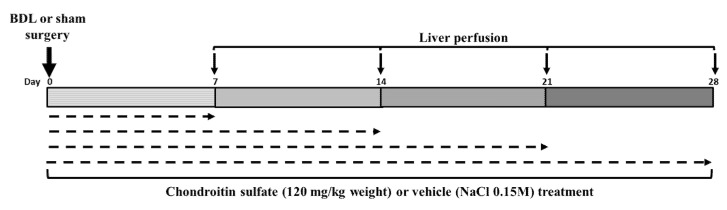
Experimental design.

Chondroitin sulfate (CS, MW 40,29 kDa) was obtained from Galena Quimica e Farmaceutica (Campinas, SP, Brazil) and consisted of a mixture of CS-A (65%). CS-C (19%) and C0S (16%). Animals were treated with CS (120 mg/kg), (BDL-CS group) or vehicle (NaCl 150 mM) (BDL group) intraperitoneally (i.p.) 24 h before the surgery, at the day of surgery (zero time), and every other day for 7, 14, 21 or 28 days. The animals were weighed at the moment that they received CS or vehicle (Figure 5).

### 4.3. Liver Perfusion

Rats were anesthetized with urethane (1.3 g/kg of body weight, i.p.), and after cannulation of the portal vein and thoracic inferior vena cava, livers were perfused with 200 mL of Krebs–Henseleit bicarbonate solution saturated with 95% O_2_ and 5% CO_2_ (pH 7.4) at 37 °C for exsanguination.

Blood was drawn from the abdominal aorta before portal vein cannulation, and serum was separated for posterior biochemical analysis. Serum activity of alanine aminotransferase (ALT) and aspartate aminotransferase (AST) was measured using Labtest Diagnostica kits (Lagoa Santa, MG, Brazil), cat No 108 and 109, respectively, in accordance with its technical manual. Using as a parameter the minimum detectable absorbance, the sensitivity photometric for each assay is 1.75 U/L, corresponding to an absorbance equal to 0.001. The inter-assay precision is 7.5% and 4.5% and for the intra assay is 1.3% and 1.6% for ALT and AST assays, respectively, in accordance with the desirable specification for total error (15–16%) and total imprecision (6.1–5.4%) based on the components of biological variation.

After exsanguination, the liver was removed, and samples were snap-frozen in liquid nitrogen or fixed in 4.5% paraformaldehyde for posterior analysis.

### 4.4. Histomorphometric Analysis of Liver Sections

Excised liver specimens were fixed in 4.5% paraformaldehyde and then embedded in paraffin. Histological analysis and measurement of collagen deposition were conducted in liver sections (4 µm) after Picrosirius (PS) staining. Semi-quantification of total collagen and parenchyma contents was performed by randomly selecting five non-overlapping areas containing portal tracts or centrilobular veins in each section at 100× magnification using an AxioImager A2 microscope (Zeiss, Oberkochen, Germany) and AxioVision 4.8 software [25]. Results are expressed as the mean percentage area of collagen and parenchyma content per field from at least 4 independent animals per group.

PS-stained liver sections were also analyzed under polarized light to differentiate type I (yellow and red) and type III (green) collagen fibers. In this process, four non-overlapping areas were randomly selected per section at 200× magnification using an Axiolab 2.0 optical microscope (Zeiss, Oberkochen, Germany). The quantification of the area occupied by the collagen fibers was performed using Image J software (NIH, Baltimore, MD, USA). Densitometry analysis data were obtained as arbitrary units between 0 and 255 [66,67]. The data are expressed as the mean ± SEM.

### 4.5. TUNEL Assay

The terminal deoxynucleotidyl transferase-mediated dUTP nick-end labeling (TUNEL) assay performed with a TACS^®^ 2 TdT Fluorescein kit (Trevigen, Gaithersburg, MD, USA) was used to quantify apoptosis in situ according to the manufacturer’s instructions. TUNEL-positive cells were counted in 10 non-overlapping microscopic fields containing portal tracts or centrilobular veins (200× magnification) per specimen using an AxioImager A2 microscope (Zeiss, Oberkochen, Germany). Results are expressed as the mean of the percentage of TUNEL-positive hepatocytes in relation to the total of hepatocytes per field.

### 4.6. Anti-PCNA Immunohistochemistry

Deparaffinized liver sections (5 µm) were subjected to heat-induced epitope retrieval (10 mM sodium citrate, 0.1% Tween 20, pH 6.0) in a microwave for 30 min. After treatment with 5% BSA and 0.1% Tween 20 on Tris-buffered saline (TBS) to reduce non-specific reactions, the liver sections were incubated with anti-proliferating cell nuclear antigen antibody (anti-PCNA, 1:50, sc-25280, Santa Cruz Biotechnology, Dallas, TX, USA) overnight at 4 °C. Following washing with TBS, the sections were incubated with anti-mouse antibody conjugated to FITC (1:100, F-0257, Sigma-Aldrich, St. Louis, MO, USA) for 2 h, and slides were mounted by addition of Fluoroshield^TM^ with DAPI (Sigma-Aldrich, St. Louis, MO, USA). In the negative control, the primary antibody was omitted. The hepatocytes with fluorescence-marked nuclei were considered positive. Results are expressed as the mean of the percentage of marked hepatocytes in relation to the total number of hepatocytes per field. The images were obtained using the AxioImager A2 microscope (Zeiss, Oberkochen, Germany) with 200× magnification. For this analysis, 10 non-overlapping microscopic fields containing portal tracts or centrilobular veins were counted per specimen from at least 5 independent animals per group.

### 4.7. Cathepsin B and Caspase-3 Activity

Liver samples were homogenized with 10 volumes of 50 mM Tris-HCl buffer pH 7.4, containing 10 mM CaCl_2_, 1 µM ZnCl_2_, 0.1% Triton X-100 and 0.25 M Sucrose. The protein concentration of homogenates was measured with a Pierce BCA Protein kit (Thermo Fisher Scientific, Waltham, MA, USA).

Cathepsin B activity was measured by incubating liver homogenates with 5 µM 2-aminobenzoyl-Gly-Ile-Val-Arg-Ala-Lys(2,4-dinitrophenol) (Abz-GIVRAK(Dnp)-OH, Aminotech, Diadema, SP, Brazil) in 75 mM Tris, 25 mM MES, 25 mM acetic acid, 25 mM glycine, 2 mM EDTA, and 2 mM dithiothreitol (pH 4.5) in a 96-well plate at 37 °C for 10 min. Fluorescence was monitored using a fluorescence spectrophotometer F-7000 (Hitachi, Tokyo, Japan), at an excitation wavelength of 320 nm and an emission wavelength of 420 nm. Results are expressed as arbitrary fluorometric units per minute per microgram of protein (AFU·min^−1^·µg^−1^) and fold-increase compared to the sham group [68].

For caspase-3 activity, liver homogenates (approximately 100 µg) were incubated with 10 µM N-Acetyl-Asp-Glu-Val-Asp-7-amido-4-Methylcoumarin (Ac-DEVD-AMC, Sigma-Aldrich, St. Louis, MO, USA) in 50 mM HEPES, 100 mM NaCl, 0.1% CHAPS, 1 mM EDTA, and 2 mM dithiothreitol (pH 7.4) in a 96-well plate at 37 °C for 60 min. Fluorescence was monitored using a fluorescence spectrophotometer F-7000 (Hitachi, Tokyo, Japan) at an excitation wavelength of 380 nm and an emission wavelength of 460 nm. Results are expressed as arbitrary fluorometric units per minute per milligram of protein (AFU·min^−1^·mg^−1^) and fold-increase compared to the sham group [69,70].

### 4.8. Statistical Analysis

Statistical analysis was performed using GraphPad Prism version 7.0. Normality of the data was verified with the Shapiro–Wilk test, and data comparison was performed using one-way ANOVA with Tukey’s multiple comparison test. The significance level for rejecting the null hypothesis was considered less than or equal to 0.05.

## 5. Conclusions

In conclusion, our study showed a promising hepatoprotective action of CS treatment during hepatic fibrogenesis induced by BDL, which promoted hepatocyte proliferation and inhibition of cell death enzymes, such as caspase and cathepsin B, resulting in preserved parenchyma content and decreased septa formation, without changing the type III to I collagen ratio in the portal triads. More studies are necessary to determine the best method of CS administration and to evaluate the action regarding liver function.

## Figures and Tables

**Table 1:** Descriptive parameters of BDL and BDL-CS rats in different induction times.

Parameters	BDL				BDL-CS			
	7d	14d	21d	28d	7d	14d	21d	28d
**N**	6	11	5	7	9	9	6	6
Age (week)	6.93 ± 0.03	7.85 ± 0.24 ^a^	7.14 ± 0.29	7.28 ± 0.18	6.65 ± 0.02	6.68 ± 0.12 ^b^	7.14 ± 0.36	7.64 ± 0.21 ^a,c^
Body weight(g)	219 ± 6	264 ± 6 ^a^	292 ± 17 ^d^	282 ± 15 ^d^	193 ± 6	213 ± 10 ^b^	290 ± 6 ^b,e^	286 ± 3 ^b,e^
Liver weight (g)	15.8 ± 0.6	21.7 ± 0.8 ^d^	26.9 ± 1.8 ^e^	24.4 ± 1.8 ^e^	13.9 ± 0.5	16.6 ± 0.8 ^b^	26.0 ± 0.9 ^b,e^	27.9 ± 1.6 ^b,e^
L/B weight ratio (%)	7.2 ± 0.2	8.2 ± 0.2	9.2 ± 0.3 ^a^	8.7 ± 0.5	7.2 ± 0.1	7.9 ± 0.4	9.0 ± 0.2 ^a^	9.8 ± 0.6 ^c,e^
ALT (U/L)	111 ± 13	149 ± 24	134 ± 24	101 ± 23	78 ± 9	105 ± 15	113 ± 16	115 ± 18
AST (U/L)	493 ± 54	522 ± 77	478 ± 42	296 ± 62	343 ± 59	447 ± 42	334 ± 49	255 ± 33

Results are mean ± SEM; ANOVA test: ^a^ *p* < 0.04 compared to the corresponding 7-day group. ^b^ *p* < 0.0007 compared to BDL 14-day group. ^c^ *p* < 0.04 compared to BDL-CS 14-day group. ^d^ *p* < 0.0060 compared to BDL 7-day group. ^e^ *p* < 0.0001 compared to the corresponding 7-day group.

## Data Availability

Data are available upon request.

## Supplementary Materials

The following are available online, Figure S1: Liver histology of BDL animals treated with vehicle (BDL group) or chondroitin sulfate (BDL-CS groups). (A–H) Representative images of the hematoxylin-eosin-stained liver sections from BDL and BDL-CS groups (1–100× magnification and 2–200× magnification; scale bar: 100 µm and 50 µm, respectively). The arrows indicate the progressive porto-portal bridging formation and the asterisks the bile ductular proliferation.
