# Supplementary material for: Chondroitin Sulfate Protects the Liver in an Experimental Model of Extra-Hepatic Cholestasis Induced by Common Bile Duct Ligation
Source: Molecules. 2022 Jan 20;27(3):654. doi: 10.3390/molecules27030654 (PMC8839946; doi:10.3390/molecules27030654)
Supplement: Supplementary file 1 [file molecules-27-00654-s001.zip › molecules-1530420-supplementary.pdf]

molecules

MDPI

Supplementary File

Chondroitin Sulfate Protects the Liver in an Experimental Model of Extra-Hepatic Cholestasis Induced by Common Bile Duct Ligation

Pedro L. R. Guedes [1], Carolina P. F. Carvalho [2], Adriana A. F. Carbonel [3], Manuel J. Simões [4], Marcelo Y. Icimoto [5], Jair A. K. Aguiar [6], Maria Kouyoumdjian [7], Marcos L. Gazarini [2] and Marcia R. Nagaoka [2,*]

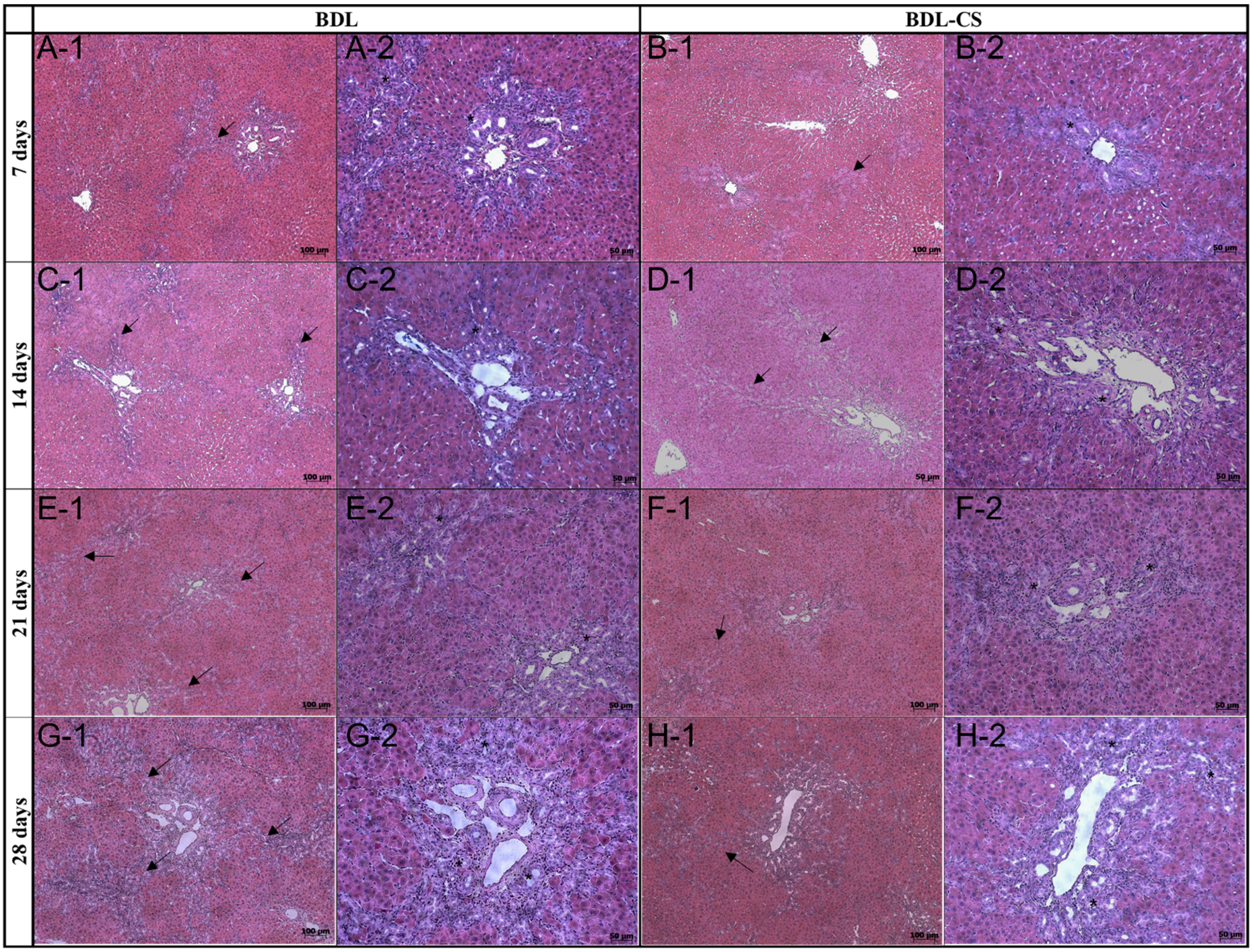

Figure S1. Liver histology of BDL animals treated with vehicle (BDL group) or chondroitin sulfate (BDL-CS groups. (A-H) Representative images of the hematoxylin-eosin-stained liver sections from BDL and BDL-CS groups (1–100× magnification and 2–200× magnification; scale bar: 100 µm and 50 µm, respectively). The arrows indicate the porto-portal bridging formation and the asterisks the bile ductular proliferation.
